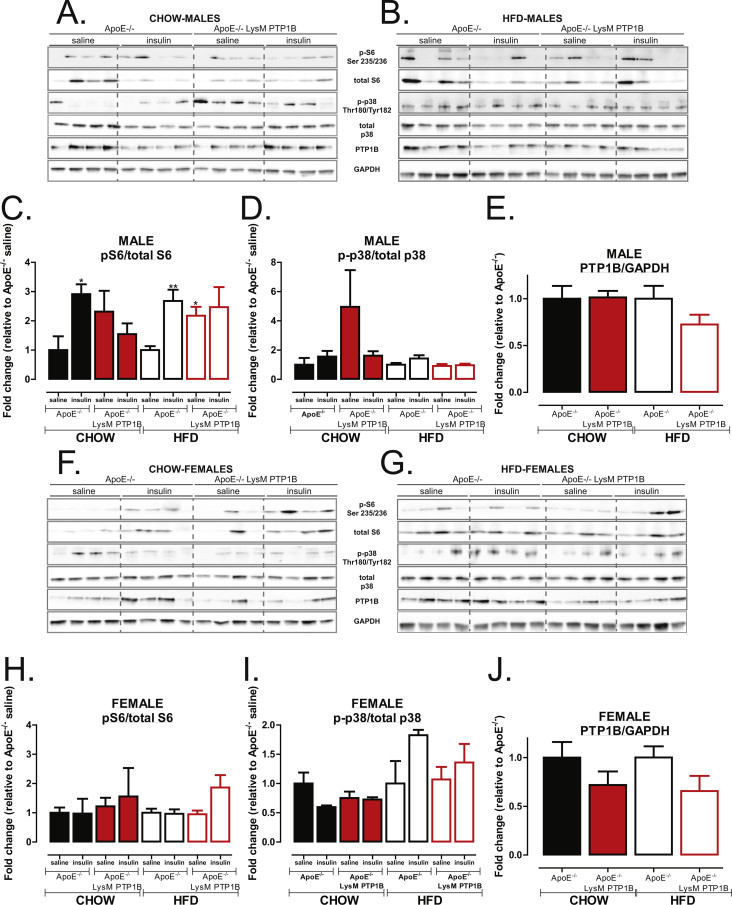# Myeloid protein tyrosine phosphatase 1B (PTP1B) deficiency protects against atherosclerotic plaque formation in the ApoE^−/−^ mouse model of atherosclerosis with alterations in IL10/AMPKα pathway

**DOI:** 10.1016/j.molmet.2017.06.003

**Published:** 2017-06-13

**Authors:** D. Thompson, N. Morrice, L. Grant, S. Le Sommer, K. Ziegler, P. Whitfield, N. Mody, H.M. Wilson, M. Delibegović

**Affiliations:** 1 Institute of Medicine, Medical Sciences and Nutrition, University of Aberdeen, Aberdeen, UK; 2 Department of Diabetes and Cardiovascular Science, University of the Highlands and Islands, Centre for Health Science, Inverness, UK

**Keywords:** PTP1B, Insulin resistance, Interleukin-10, AMPK, Atherosclerosis

## Abstract

**Objective:**

Cardiovascular disease (CVD) is the most prevalent cause of mortality among patients with Type 1 or Type 2 diabetes, due to accelerated atherosclerosis. Recent evidence suggests a strong link between atherosclerosis and insulin resistance due to impaired insulin receptor (IR) signaling. Moreover, inflammatory cells, in particular macrophages, play a key role in pathogenesis of atherosclerosis and insulin resistance in humans. We hypothesized that inhibiting the activity of protein tyrosine phosphatase 1B (PTP1B), the major negative regulator of the IR, specifically in macrophages, would have beneficial anti-inflammatory effects and lead to protection against atherosclerosis and CVD.

**Methods:**

We generated novel macrophage-specific PTP1B knockout mice on atherogenic background (ApoE^−/−^/LysM-PTP1B). Mice were fed standard or pro-atherogenic diet, and body weight, adiposity (echoMRI), glucose homeostasis, atherosclerotic plaque development, and molecular, biochemical and targeted lipidomic eicosanoid analyses were performed.

**Results:**

Myeloid-PTP1B knockout mice on atherogenic background (ApoE^−/−^/LysM-PTP1B) exhibited a striking improvement in glucose homeostasis, decreased circulating lipids and decreased atherosclerotic plaque lesions, in the absence of body weight/adiposity differences. This was associated with enhanced phosphorylation of aortic Akt, AMPKα and increased secretion of circulating anti-inflammatory cytokine interleukin-10 (IL-10) and prostaglandin E2 (PGE_2_), without measurable alterations in IR phosphorylation, suggesting a direct beneficial effect of myeloid-PTP1B targeting.

**Conclusions:**

Here we demonstrate that inhibiting the activity of PTP1B specifically in myeloid lineage cells protects against atherosclerotic plaque formation, under atherogenic conditions, in an ApoE^−/−^ mouse model of atherosclerosis. Our findings suggest for the first time that macrophage PTP1B targeting could be a therapeutic target for atherosclerosis treatment and reduction of CVD risk.

## Highlights

• PTP1B inhibition as therapy for atherosclerosis/cardiovascular disease is proposed.
• Myeloid-PTP1B mice on ApoE^−/−^ background (ApoE^−/−^/LysM-PTP1B) were generated.
• ApoE^−/−^/LysM-PTP1B had improved glucose homeostasis with no body weight differences.
• ApoE^−/−^/LysM-PTP1B had lower lipids and protection against atherosclerotic plaques.
• Protection was via a PGE_2_/IL-10/AMPKα mechanism.

## 1 Introduction

Despite extensive research and major advancements in modern medicine, CVDs are still the leading cause of death worldwide accounting for 17.3 million deaths globally per year (WHO Statistics, http://www.who.int/mediacentre/factsheets/fs317/en/). Such diseases are exacerbated by obesity and secondary pathologies such as Type 2 diabetes and atherosclerosis [1]; therefore, it is not surprising that the number of deaths attributed to CVDs is expected to rise within the next decade. Atherosclerosis is an inflammatory disease of the large and medium-sized arteries, and it is typically characterized by formation of lesions, which can lead to arterial thrombosis and ischemic injury. The proposed mechanisms that link accelerated atherosclerosis and increased cardiovascular risk in diabetic and obese patients are poorly understood. It has been suggested that an association between hyperglycaemia and intracellular metabolic changes can result in oxidative stress, low-grade inflammation, and endothelial dysfunction [2]. Insulin resistance has also been implicated in the pathogenesis of atherosclerosis adding a further layer of complexity [2]. A better understanding of the mechanisms controlling these diseases is needed, and more targeted and effective therapeutics are urgently sought.

In mice, deletion of the insulin receptor (IR) from vascular endothelial cells in the apolipoprotein-E-deficient mice (ApoE^−/−^) leads to an increased rate of atherosclerosis development [3]. Moreover ApoE^−/−^ mice with a heterozygous deletion of the IR and its downstream target, Insulin Receptor Substrate 1 (IRS1), also develop accelerated atherosclerosis [4], as well as mice lacking insulin receptor substrate 2 (IRS2^−/−^) [5]. Furthermore, decreased insulin signaling in non-hematopoietic cells, as achieved by transplantation of ApoE^−/−^ mouse model of atherosclerosis with bone marrow cells from IRS1^+/−^ IR^+/−^ ApoE^−/−^ mice, contributed to increased atherogenesis in these mice [4]. In all these disorders, macrophages play a key role driving the pathology [6], [7], [8], [9]; therefore, targeting their activity could prove effective.

Protein tyrosine phosphatase (PTP)1B has been identified as the major negative regulator of the IR itself [10]. In mice, whole body PTP1B^−/−^ studies established PTP1B as a major regulator of insulin sensitivity and body mass, via inhibition of insulin and leptin signaling, respectively [11], [12]. Our recent research found that deletion of protein tyrosine phosphatase 1B (PTP1B) within the myeloid cell lineage led to protection against high-fat diet (HFD)-induced and bacterial toxin (lipopolysaccharide LPS)-induced inflammation and hyperinsulinemia, due to increased anti-inflammatory IL-10 and decreased pro-inflammatory TNFα cytokine secretion *in vivo* [13]. Therefore, we hypothesized that inhibiting the activity of PTP1B specifically in macrophages, through genetic deletion, would have beneficial anti-inflammatory effects and lead to protection against atherosclerosis. To assess this, we used the ApoE^−/−^ mouse model of atherosclerosis under physiological and atherogenic conditions.

## 2 Research design and methods

### 2.1 Animal studies

All animal procedures were performed under a project license approved by the U.K. Home Office under the Animals (Scientific Procedures) Act 1986 (PPL 60/3951). ApoE^−/−^ mice were bred in house, and crosses were generated by breeding ApoE^−/−^ to mice devoid of myeloid PTP1B (PTP1B-LysMCre as previously described [14]) to generate ApoE^−/−^/LysM-PTP1B. At 12 weeks of age, male and female ApoE^−/−^ and ApoE^−/−^/LysM-PTP1B mice were group housed (n = 3–6 per cage) and placed on either chow or high fat diet (HFD 42% from fat, 0.2% cholesterol, Envigo, Huntingdon UK) for 12 weeks. Mice were maintained at 22–24 °C on 12-h light/dark cycle with free access to food/water. At week 12, mice were fasted for 5 h and injected with either saline or insulin (10 mU/g body weight) for 10 min prior to culling by CO_2_ inhalation and subsequent cervical dislocation. Heart and aortic tissues were harvested and collected for further analysis. Tissues for subsequent western blot or qPCR analysis were frozen in liquid nitrogen and stored at −80 °C until needed, whereas tissues for histology were immersed in formalin for 24 h at 4 °C, then stored at 4 °C in PBS until analyzed.

### 2.2 Glucose and insulin tolerance tests

Mice were fasted for 5 h prior to commencement of glucose or insulin tolerance tests (GTT and ITT, respectively). Briefly, baseline glucose levels were sampled from tail blood using glucose meters (AlphaTRAK, Abbott Laboratories, Abbot Park, IL, USA). Subsequently mice were injected I.P. with 20% glucose (w/v) or insulin (0.75 mU/g body weight) and blood glucose measured at 15, 30, 60, and 90 min post-injection.

### 2.3 Body fat mass composition

The body composition of each mouse was analyzed using an Echo MRI-3-in-1 scanner (Echo Medical Systems, Houston, TX, USA). Mice were placed individually into a plastic tube and inserted into the scanner where total body fat and lean mass were determined.

### 2.4 Isolation and maturation of bone marrow derived macrophages

Bone marrow derived macrophages (BMDMs) were isolated and cultured as previously described [13]. Briefly, following cervical dislocation, the bone marrow was flushed from the femurs and tibiae using sterile phosphate buffered saline (PBS, Gibco) to disrupt the bone marrow plugs and passed through a cell strainer. The cells were then centrifuged at 1800*g* for 5 min and resuspended in red blood cell lysis buffer before the reaction quenched with PBS and respun to pellet cells. Cells were counted and seeded onto 6 well plates, at a density of 2 million per well, and cultured in complete media (DMEM (Gibco), 10% FBS (Invitrogen, Paisley, UK), 100 U/ml penicillin (Gibco) and 100 mg/ml streptomycin (Gibco)) supplemented with 20% L929-cell conditioned medium) for 6 days (media was replaced on day 3) and maintained at 37 °C in a humidified atmosphere with 5% CO_2_. Mature macrophages were harvested on day 6 for subsequent experiments.

### 2.5 Immunoblotting

Frozen aorta tissues were homogenized in 300 μl of ice-cold Radioimmunoprecipitation assay (RIPA) buffer (10 mM Tris–HCl pH 7.4, 150 mM NaCl, 5 mM EDTA pH 8.0, 1 mM NaF, 0.1% SDS, 1% Triton X-100, 1% Sodium Deoxycholate with freshly added 1 mM NaVO_4_ and protease inhibitors) using a PowerGen 125 homogenizer and lysates normalized to 1 μg per 1 μl. Proteins were separated on a 4–12% Bis-Tris gel by SDS-PAGE and transferred onto nitrocellulose membrane. Membranes were probed for the following; p-IR (Tyr 1162/1163), IR β-chain, p-AKT (Ser 473), p-p38 (The 181/Tyr 182), total p-38 p-S6 (Ser 235/236), total S6, p-AMPKα (Thr 172), total AMPKα, PTP1B and GAPDH.

### 2.6 Histology

Immediately following cervical dislocation, hearts were immersed in formalin and stored at 4 °C for 24 h before being transferred to PBS until further analysis. Hearts were bisected to remove the lower ventricles, frozen in OCT and subsequently sectioned at 5 μm intervals until the aortic sinus was reached. Sections were mounted and stained with oil red O to assess plaque formation. Images were captured using a light microscope and plaque formation quantified using Image J software.

### 2.7 Serum analysis

Blood was collected during terminal procedures after fasting (5 h) and spun to isolate serum, then stored at −80 °C. Serum samples were subsequently analyzed for insulin and IL-10 levels using ELISAs (Millipore and R&D Systems, respectively) or total cholesterol and triglycerides (Sigma). For eicosanoid analysis, PGE_2_ was extracted from serum in methanol and isolated by C18 solid-phase extraction chromatography. PGE_2_-d_4_ (Cayman Chemicals, Ann Arbor, MI, USA) was added to the experimental system as an internal standard. PGE_2_ was analyzed by liquid chromatography-tandem mass spectrometry (LC-MS/MS) using a TSQ Quantum Ultra triple quadrupole mass spectrometer coupled to an Accela 1250 UHPLC system (Thermo Scientific, Hemel Hempstead, UK) in negative ion mode with a C18 column and methanol-water gradient. Quantification was performed using the multiple reaction monitoring (MRM) mode using the transitions PGE_2_ (351 → 271) and PGE_2_-d_4_ (355 → 193). The concentration of PGE_2_ was determined by comparison to a calibration curve run in parallel.

### 2.8 Statistical analysis

We expressed all values as mean ± S.E.M. We determined group sizes by performing a power calculation to lead to an 80% chance of detecting a significant difference (p ≤ 0.05). For both *in vivo* and *ex vivo* data, each *n* value corresponds to a single mouse. Statistical analyses were performed by using one-way or Two-way ANOVA followed by Tukey's or Dunnett's multiple-comparison tests to compare the means of three or more groups or by an unpaired two-tailed Student's *t*-test to compare the means of two groups. Variances were similar between groups. In all figures, */^#^p ≤ 0.05, **/^##^p ≤ 0.01, ***/^###^p ≤ 0.001, ****p ≤ 0.0001. All analyses were performed using GraphPad Prism (GraphPad Software).

## 3 Results

### 3.1 Myeloid specific PTP1B knockout mice exhibit improved glucose homeostasis independently of alterations in body weight/adiposity

**Figure 1:**
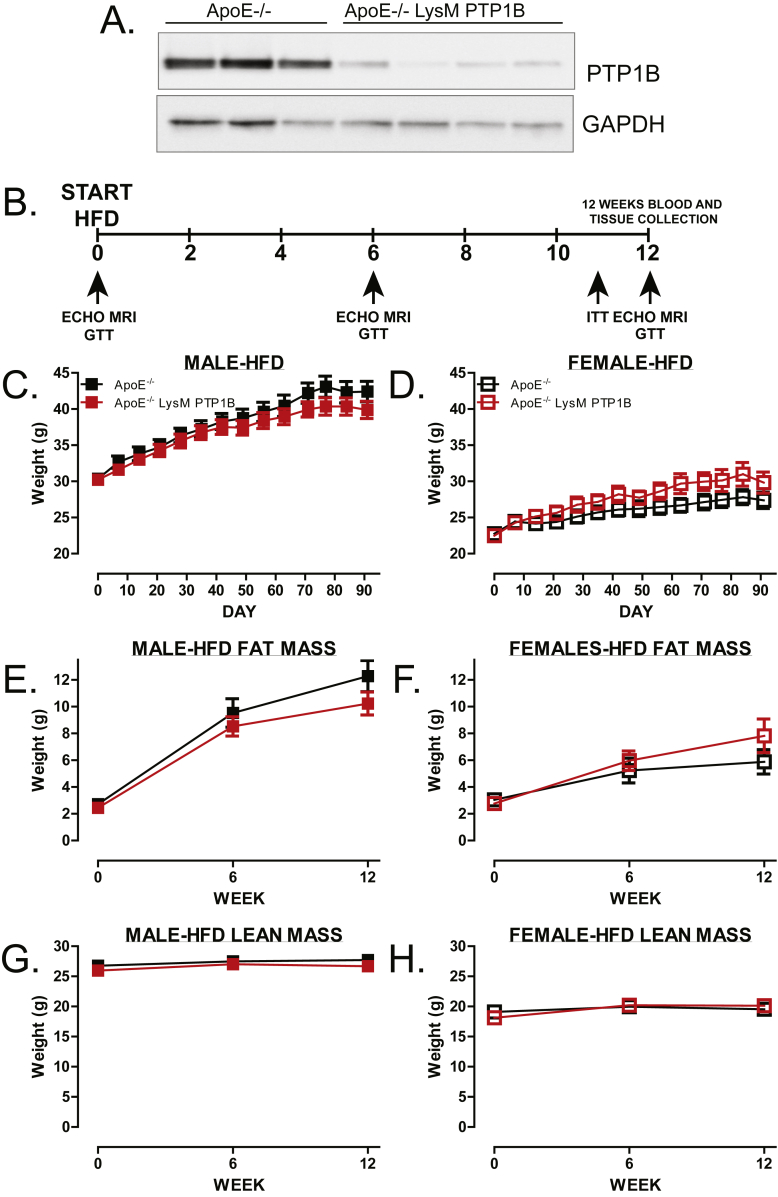
**Myeloid specific PTP1B deletion does not affect adiposity**. (**A**) Western blot of bone marrow derived macrophages depicting deletion of PTP1B in LysM-PTP1B/ApoE^−/−^ mice, GAPDH was used as a loading control. (**B**) Schematic representation of the experimental design. Mice were divided into ApoE^−/−^ or LysM-PTP1B/ApoE^−/−^ groups, both males and females were tested (n = 9–13 per group). Weekly body weights of male (**C**) and female (**D**) cohorts. Body composition was analyzed using an Echo MRI-3-in-1 scanner where total body fat (**E, F**) or lean mass (**G, H**) was determined (n = 9–11 per group). Data are represented as mean ± S.E.M. and analyzed by Two-way ANOVA followed by Bonferonni multiple comparison *t*-tests.

We recently demonstrated that myeloid PTP1B (LysM PTP1B) deletion protected against LPS-induced inflammation and led to increased production of the anti-inflammatory cytokine interleukin 10 (IL-10) [14]. This intriguing finding compliments numerous studies implicating IL-10 as having a protective role in atherogenesis and CVD (reviewed in [15]). Given the importance of myeloid cells, particularly macrophages, in the pathogenesis of both atherosclerosis and insulin signaling, we sought to determine if PTP1B deletion in these cells would have beneficial protective effects against atherosclerosis development. To achieve this, we generated novel myeloid-specific PTP1B knockout mice using an atherogenic mouse model (ApoE^−/−^/LysM-PTP1B) to address this. These mice developed normally and did not display any defects. To confirm myeloid deletion, BMDMs from ApoE^−/−^ and ApoE^−/−^/LysM-PTP1B were harvested and probed for protein expression of PTP1B (Figure 1A).

**Figure 2:**
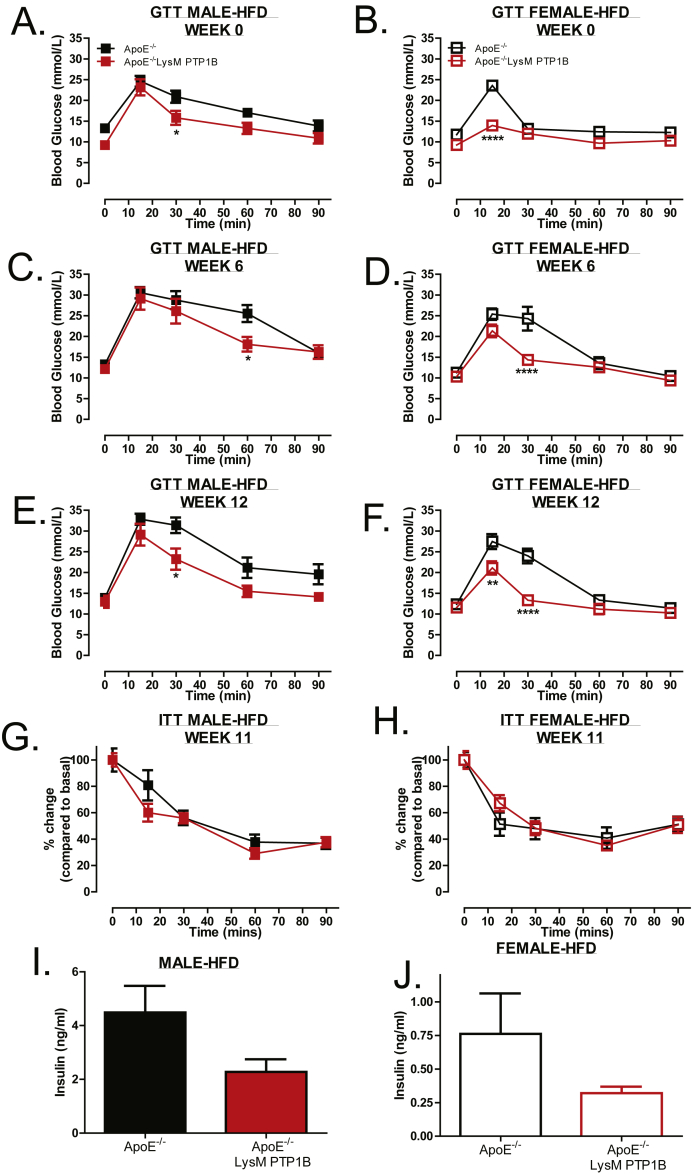
**Myeloid specific PTP1B deletion improves glucose maintenance independently of weight gain in HFD fed and CHOW fed mice**. Glucose tolerance tests at week 0, 6 and 12 of male (**A, C, E**) and female (**B, D, F**) mice fed HFD and insulin tolerance tests (**G, H,** male and female, respectively) at week 11. Mice were fasted for 5 h prior to basal glucose monitoring (as described in materials and methods) and subsequently mice injected I.P. with 20% glucose (w/v) or insulin (0.75 mU/g body weight) and blood re-analyzed at 15, 30, 60 and 90 min post-injection. Blood was collected at terminal culls and serum analyzed for circulating insulin levels (**I, J**) using ELISA (Millipore). Data are represented as mean ± S.E.M. (n = 7 per group) and analyzed by unpaired two-tailed *t*-tests or two-way ANOVA followed by Bonferroni multiple comparison *t*-tests where *p ≤ 0.05, **p ≤ 0.01, ****p ≤ 0.0001 when compared to ApoE^−/−^ control groups.

Myeloid-specific PTP1B knockout mice on an atherogenic background (LysM-PTP1B/ApoE^−/−^) were placed on HFD for 12 weeks or chow as a control. During this time, weight gain, body composition, glucose tolerance, and insulin tolerance were assessed in both male and female mice from each genotype (Figure 1B). In contrast to global PTP1B deletion [11], there were no differences in weight gain or body composition between genotypes in either, male or female mice fed a HFD (Figure 1 C, E, G and Figure 1 D, F, H respectively) or chow (Supplemental Figure 1 A, B). As expected, male mice weighed more than females with the greatest differences seen in lean mass (Figure 1 G, H), rather than fat mass (Figure 1 E, F), and gained more weight over time. Despite no differences in weight gain, ApoE^−/−^/LysM-PTP1B animals exhibited markedly improved glucose tolerance when compared to ApoE^−/−^, which was more pronounced in the female mice (Figure 2 A, B). This improved glucose handling was maintained at 6 (Figure 2 C, D) and 12 weeks (Figure 2 E, F) of HFD and again more pronounced in females. When aged, 6 month old ApoE^−/−^/LysM-PTP1B chow fed mice still exhibited improved glucose handling when compared to ApoE^−/−^ mice (Supplemental Figure 1C, D), in the absence of body weight changes (Supplemental Figure 1 A, B). Moreover, there was no difference between genotypes in HFD cohorts when subjected to insulin tolerance tests (Figure 2 G, H), nor in circulating insulin levels (Figure 2 I, J). The lack of difference in circulating insulin levels was also observed in chow-fed mice regardless of gender or genotype (Supplemental Figure 1 E, F). Given these data, the improved glucose homeostasis could not be attributed to differences in weight/adiposity and instead due to myeloid-specific PTP1B deficiency.

### 3.2 Myeloid PTP1B deficiency on ApoE^−/−^ background results in decreased cholesterol and triglyceride levels and protection against diet-induced increase in atherosclerotic plaque area

**Figure 3:**
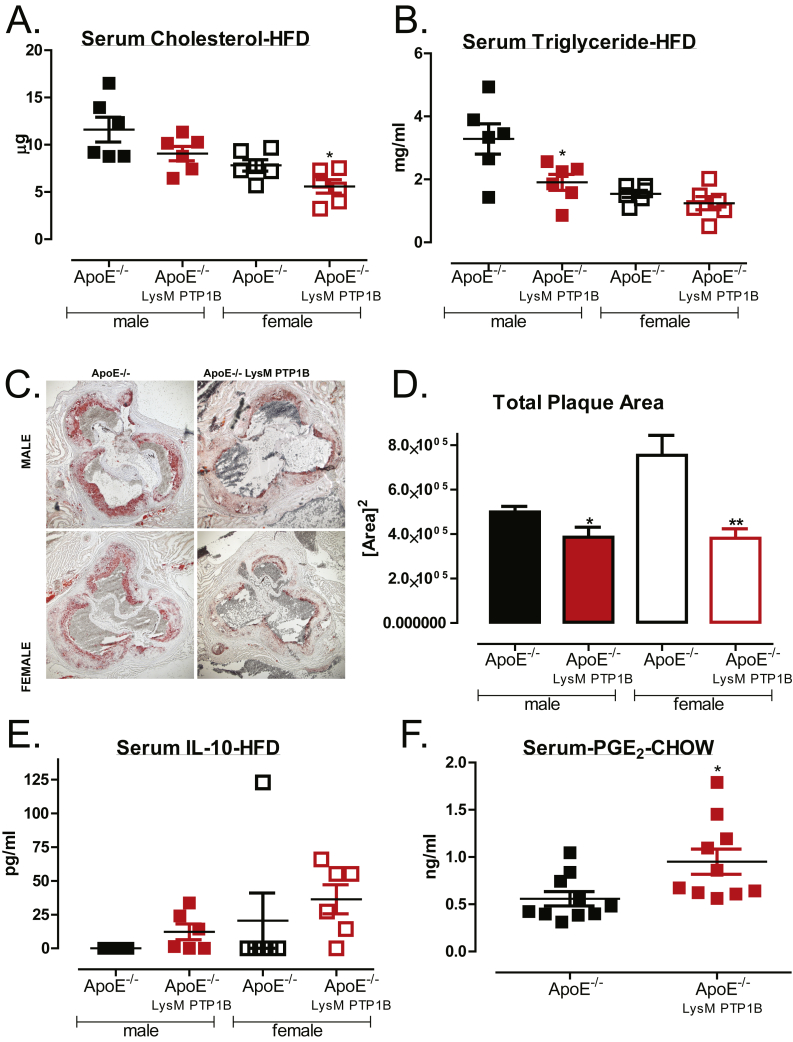
**Myeloid specific PTP1B deletion reduces serum cholesterol and triglycerides and reduces atherosclerotic plaque formation**. Blood was collected at terminal culls and serum analyzed for circulating total cholesterol (**A**) triglyceride levels (**B**) using ELISA (Millipore) (**C**) Representative (n = 5–6 per group) aortic root sections stained with Oil Red O. (**D**) Quantification of plaque area as analyzed using Image J software. Serum analysis of circulating IL-10 under HFD-feeding conditions (**E**) and PGE_2_ under chow-feeding conditions (**F**) quantified by ELISA and lipidomics, respectively. For IL-10 analysis, values below the level of detection were designated 0. Data are represented as mean ± S.E.M. and analyzed by one-way ANOVA, followed by unpaired two-tailed *t*-tests where *p ≤ 0.05 and **p ≤ 0.01, when compared to ApoE^−/−^ control groups.

Plaque formation in atherosclerosis is associated with lipid accumulation within the vessels and arterial walls. Current treatments include the use of lipid lowering drugs such as statins, which serve to reduce levels within the blood. We found that total serum cholesterol was decreased in ApoE^−/−^/LysM-PTP1B female mice in both, HFD (Figure 3A) and chow-fed (Supplemental Figure 2A) cohorts, whereas triglyceride levels were significantly decreased in female chow-fed (Supplemental Figure 2B) and male HFD-fed (Figure 3B) ApoE^−/−^/LysM-PTP1B mice.

The ultimate goal of this study was to test whether myeloid PTP1B deficiency was sufficient to protect against atherosclerotic plaque formation. To assess this, sections of aorta, beginning at the aortic root, were mounted and stained with Oil Red O. This revealed that there was significantly less plaque formation in ApoE^−/−^/LysM-PTP1B mice when compared to ApoE^−/−^ HFD mice, both in male and female mice, although more prominent in females (Figure 3C, D). Therefore, we demonstrate for the first time that myeloid-PTP1B deficiency alone is sufficient to prevent atherosclerotic plaque formation under obesogenic, HFD-feeding condition, on an atherogenic ApoE^−/−^ background.

### 3.3 Myeloid PTP1B deficiency on ApoE^−/−^ background results in increased PGE_2_ and IL-10 secretion

Since we initially hypothesized that increased IL-10 levels associated with myeloid-PTP1B deficiency may create an anti-inflammatory environment and lead to protection against atherosclerosis development, we next determined circulating levels of IL-10 in ApoE^−/−^/LysM-PTP1B and ApoE^−/−^ controls on chow and HFD. In agreement with our hypothesis, whilst only one of the ApoE^−/−^ controls had any detectable circulating IL-10 serum levels, IL-10 secretion was increased in ApoE^−/−^/LysM-PTP1B, male and female, HFD-fed mice (Figure 3E). Similar data was observed in the aged chow-fed ApoE^−/−^/LysM-PTP1B females (Supplementary Fig. 2C). In addition, when male and female data were combined, chow-fed ApoE^−/−^/LysM-PTP1B exhibited a significant increase in circulating levels of the eicosanoid, prostaglandin E2 (PGE_2_) (Figure 3F), which has been recently implicated as essential in switching macrophages from a pro-inflammatory M1 to an anti-inflammatory M2 phenotype by stimulating IL-10 release [16], [17]. Although not significant, a similar trend was observed when the groups were separated by gender (Supplemental Fig. 2D). In keeping with a decrease in pro-inflammatory signaling, we investigated the expression of genes important in the inflammatory response. More specifically, atherosclerotic plaque development is catalyzed by an infiltration of leukocytes to the aortic tissues. Such events are facilitated by monocyte chemoattractant protein-1 (MCP-1) and adhesion molecules including intracellular cell adhesion molecule-1 (ICAM-1) and vascular cell adhesion molecule-1 (VCAM-1), that serve to recruit monocytes and facilitate their transmigration into the vasculature, respectively [5]. To assess whether changes in the expression of these genes within ApoE^−/−^/LysM-PTP1B mice could account for the decrease in plaque formation, we determined levels in aortic tissue from our mouse groups (Supplemental Figure 2). While there were no differences in aortic MCP-1 expression levels, ICAM levels were higher in male HFD-fed ApoE^−/−^/LysM-PTP1B mice in comparison to chow-fed ApoE^−/−^/LysM-PTP1B mice (Supplemental Figure 3A), whilst VCAM-1 levels were upregulated in HFD-fed male ApoE^−/−^/LysM-PTP1B mice in comparison to ApoE^−/−^ mice (Supplemental Figure 3C). In contrast, no significant increases were seen in the female ApoE^−/−^/LysM-PTP1B mice, suggesting a less pro-inflammatory environment in female mice, which could explain the more pronounced reduction in plaque formation when compared to female ApoE^−/−^ controls.

### 3.4 Myeloid PTP1B deficiency on ApoE^−/−^ background is associated with hyperphosphorylation of Akt and AMPKα1 in the aorta

**Figure 4:**
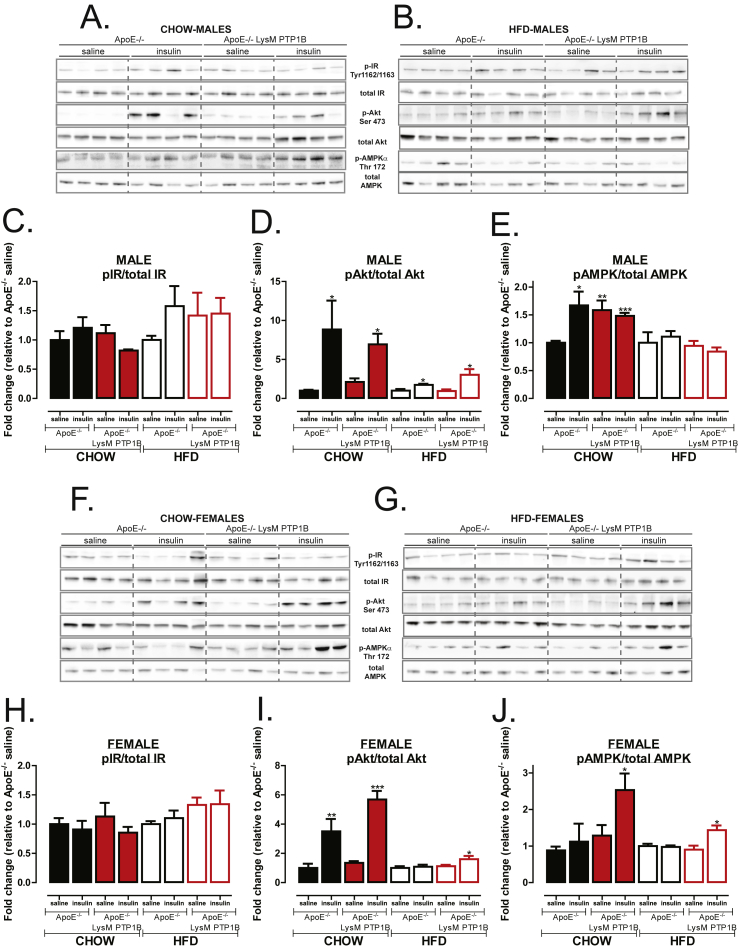
**Myeloid specific PTP1B deletion improves Akt and AMPK signaling in CHOW-fed and HFD-fed mice** (**A, B, F, G**) Western blot analysis of aortic tissues from male (**A, B**) and female (**F, G**) ApoE^−/−^ or LysM-PTP1B/ApoE^−/−^ mice fed either chow (**A, F**) or HFD (**B, G**) and injected with either saline or insulin immediately prior to culling. Quantification of p-IR (Tyr 1162/1163) (**C, H**), p-Akt (Ser 473) (**D, I**) and p-AMPKα (Thr 172) (**E, J**) of male and females represented in **A**, **B** and **F**, **G** respectively. Data are represented as mean ± S.E.M. and analyzed by one way ANOVA followed by Dunnets post hoc analysis or unpaired two-tailed *t*-tests where *p ≤ 0.05, **p ≤ 0.01, ***p ≤ 0.01 when compared to saline or ApoE^−/−^ control groups.

Previous studies using ApoE^−/−^ mice heterozygous for both IR and the insulin receptor substrate-1 (IRS1) protein found that the apolipoprotein-E deficiency resulted in increased atherosclerosis development [4]. A similar phenotype was noted when IRS2 was deleted within the same ApoE^−/−^ background [5]. Given these data, we hypothesized that an upregulation of IR signaling and associated downstream pathways could account for the beneficial effects of PTP1B deletion in our study. Therefore, we investigated the IR signaling pathway in aortic tissues. In contrast to our hypothesis, there were no detectable differences in phosphorylation of the IR itself in aortic tissue from either chow-fed (Figure 4 A, C, F, H) or HFD-fed (Figure 4 B, C, G, H) ApoE^−/−^/LysM-PTP1B and ApoE^−/−^ mice. However, there was a striking enhancement of downstream signaling, as indicated by hyperphosphorylation of Akt, in both male and female ApoE^−/−^/LysM-PTP1B mice (Figure 4 A, B, D & F, G, I). As expected, HFD-feeding resulted in a blunted response to insulin-stimulated Akt phosphorylation in both males (Figure 4D) and females (Figure 4I), and in females, insulin was able to stimulate Akt phosphorylation only in ApoE^−/−^/LysM-PTP1B mice (Figure 4I). Male ApoE^−/−^/LysM-PTP1B mice also exhibited enhanced pS6 signaling (Supplemental Figure. 3 A, B, C & F, G, H). Considering that IL10 has recently been shown to directly phosphorylate and activate AMPKα [18] and that IL-10 secretion is upregulated in ApoE^−/−^/LysM-PTP1B mice, we postulated that these mice may exhibit alterations in AMPKα activation. Consistent with this notion, AMPKα activation in male chow fed aortic tissue was upregulated under basal condition in ApoE^−/−^/LysM-PTP1B mice (Figure 4 A, E), and pAMPKα signaling was enhanced in female chow and HFD-fed insulin-stimulated ApoE^−/−^/LysM-PTP1B mice (Figure 4 A, B, E & F, G, J). These data suggest that myeloid-PTP1B deficiency induces AMPK activation. We observed no differences in p38 phosphorylation across genotype or gender (Supplemental Figure 4 A, B, F, G). Furthermore, there were no differences in aortic-PTP1B protein levels, as expected and consistent with myeloid-specific PTP1B deficiency (Supplemental Figure 3 E, J). Therefore, since the deletion is limited to the myeloid cell lineage, the increase in aortic Akt and AMPKα phosphorylation can be attributed solely to myeloid-specific PTP1B deficiency.

## 4 Discussion

In conclusion, we demonstrate here that myeloid-specific genetic deletion of PTP1B in the ApoE^−/−^ mouse model of atherosclerosis leads to protection against atherosclerosis development, suggesting beneficial effects of PTP1B inhibition for the reduction of cardiovascular risk and treatment of CVDs. We present evidence that myeloid-PTP1B deficiency results in activation of aortic Akt and AMPKα1, without measurable alterations in insulin receptor phosphorylation. We also demonstrate that myeloid-specific PTP1B deficiency on an ApoE^−/−^ background leads to improved glucose maintenance, independently of body weight/adiposity differences, and that this results in increased circulating levels of the anti-inflammatory cytokine IL-10, and the vasodilator eicosanoid PGE_2_. Finally, we present evidence, for the first time, that demonstrates that myeloid-PTP1B targeting results in a decrease in circulating serum cholesterol (female mice under chow and HFD-feeding conditions) and triglyceride levels (female mice on chow diet and male mice on HFD diet) and protection against atherosclerotic plaque formation.

Our data compliment a recent study by Kandadi et al. [19] that found whole body PTP1B deletion improved cardiomyocyte contractility in mice fed HFD, through an AMPK-dependent mechanism. Furthermore, myeloid-specific deletion of AMPKα1 on an LDLR^−/−^ mouse background, resulted in hypercholesterolemia, increased macrophage inflammation and plaque infiltration and exacerbated atherogenesis [20]. Thus, the increase we observe in aortic AMPKα1 phosphorylation with myeloid-PTP1B deficiency, and associated protection against atherosclerotic plaque accumulation, suggest that PTP1B inhibition may be anti-atherogenic through an AMPKα1-driven mechanism and warrants investigation in future studies.

In further support of PTP1B inhibition as a potential therapeutic target for atherosclerosis, we have previously demonstrated that hepatic-PTP1B deficiency was sufficient to protect against HFD-induced endothelial dysfunction, in the absence of any changes in body mass/adiposity [21]. Considering that endothelial dysfunction acts as an independent predictor of cardiovascular events, this was firm evidence that PTP1B inhibition may not only play an anti-diabetic role, but also hold potential for treatment of CVDs.

Critically, atherosclerosis is now well regarded as a chronic inflammatory disease with current anti-inflammatory therapeutics aimed at targeting specific parts of the cascade [22]. Moreover, recent studies have focused on macrophages and, in particular, promotion from pro-inflammation to pro-resolution, as a means to reduce atherogenesis and similar chronic inflammatory pathologies [23], [24]. Indeed, the anti-inflammatory cytokine IL-10, which promotes resolution by stimulating efferocytosis and tissue repair, is now being engineered as nanoparticles to allow more specific targeting, and has resulted in the stabilization of the fibrous cap and necrotic core reduction in HFD-fed LDLR^−/−^ mice [25]. Furthermore, a recent study by Zhu et al. [18] found that IL-10 stimulation of AMPKα phosphorylation and subsequent downstream PI3K/Akt/mTORC1 signaling was critical for eliciting the anti-inflammatory properties of this cytokine. In support of these data, a previous study from our lab found myeloid-deletion of PTP1B resulted in increased anti-inflammatory signaling, as evidenced by a decrease in pro-inflammatory IL-6 and TNFα, and an increase in pro-resolution IL-10 [14].

Our targeted lipidomic eicosanoid analysis revealed a specific increase in circulating PGE_2_ levels; an eicosanoid essential in eliciting the pro-inflammatory cascade. In contrast to its role as a pro-inflammatory molecule, recent research has implicated PGE_2_ to behave as an immunomodulator, acting on four distinct G-protein coupled receptors (GPCRs, EP1-4) to both, inhibit and stimulate, the synthesis of pro- and anti-inflammatory cytokines, respectively. Importantly, PGE_2_ has been found to be essential in switching macrophages from a pro-inflammatory M1 to an anti-inflammatory M2 phenotype and does so through an IL-10 dependent mechanism [16], [17]. In addition, deletion of PTP1B enhances the expression of COX2, which is upstream of PGE_2_ release [26], and was found to be protective against endothelial dysfunction in a model of Type 1 diabetes. Finally, PGE_2_ has been implicated in providing a protective role in diet-induced obesity and metabolic syndrome, critical for white to brown fat conversion [27].

In conclusion, the data presented herein, demonstrate that aortic Akt and AMPKα signaling was enhanced in response to genetic deletion of myeloid-PTP1B on ApoE^−/−^ background, and accompanied by increased circulating levels of the anti-inflammatory cytokine IL-10 and the vasodilator eicosanoid, PGE_2_. Given these findings, the beneficial effects of PTP1B inhibition on the atherogenesis process is likely to be through an IR-independent, PGE2/IL-10/AMPKα-driven mechanism. Therefore, based on our data, we propose that PTP1B inhibitors, currently in pre-clinical trials for Type 2 diabetes and breast cancer [28], could be repurposed for the treatment of atherosclerosis and reduction of CVD risk.

## Author contributions

DT performed the experiments and wrote the manuscript, N Morrice, assisted with GTT experiments and terminal culls, LG optimized the qPCR. N. Mody and SLM aided with terminal procedures. H.M.W suggested experiments and reviewed the manuscript. KW and PW performed and analyzed the PGE_2_ lipidomics. MD conceived and designed the experiments and wrote the manuscript.

## Figures and Tables

**Figure:**
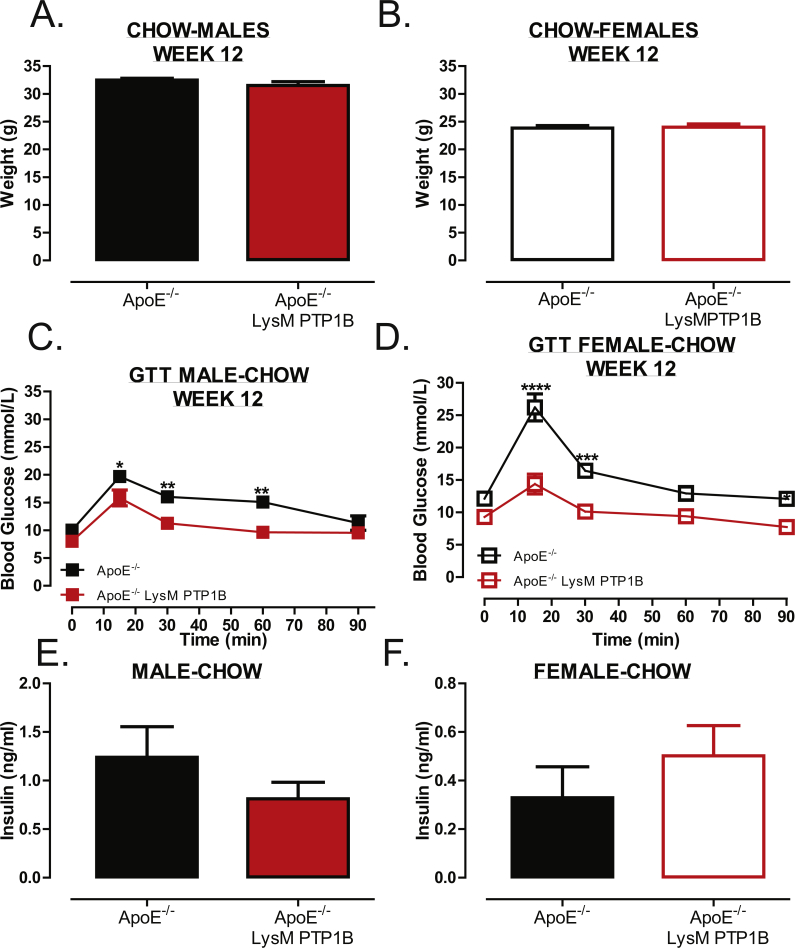


**Figure:**
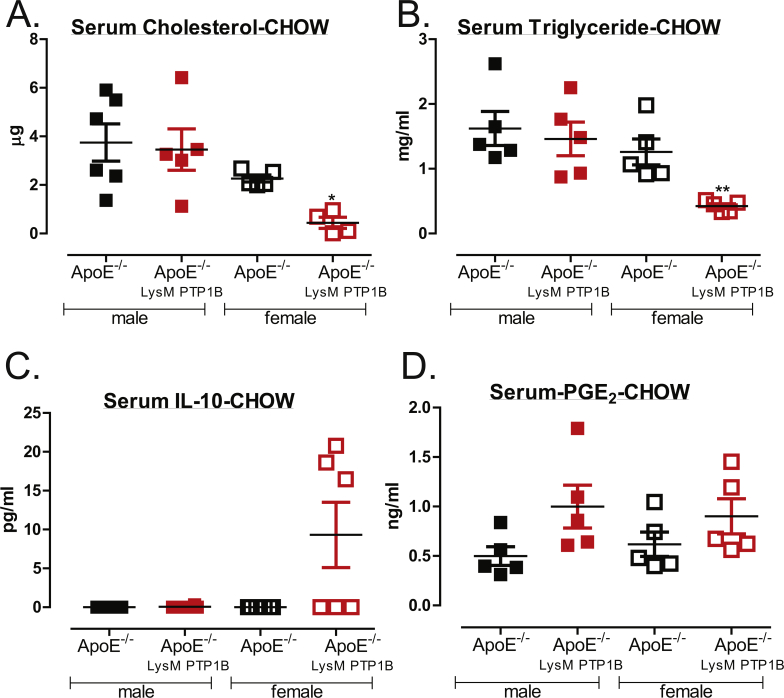


**Figure:**
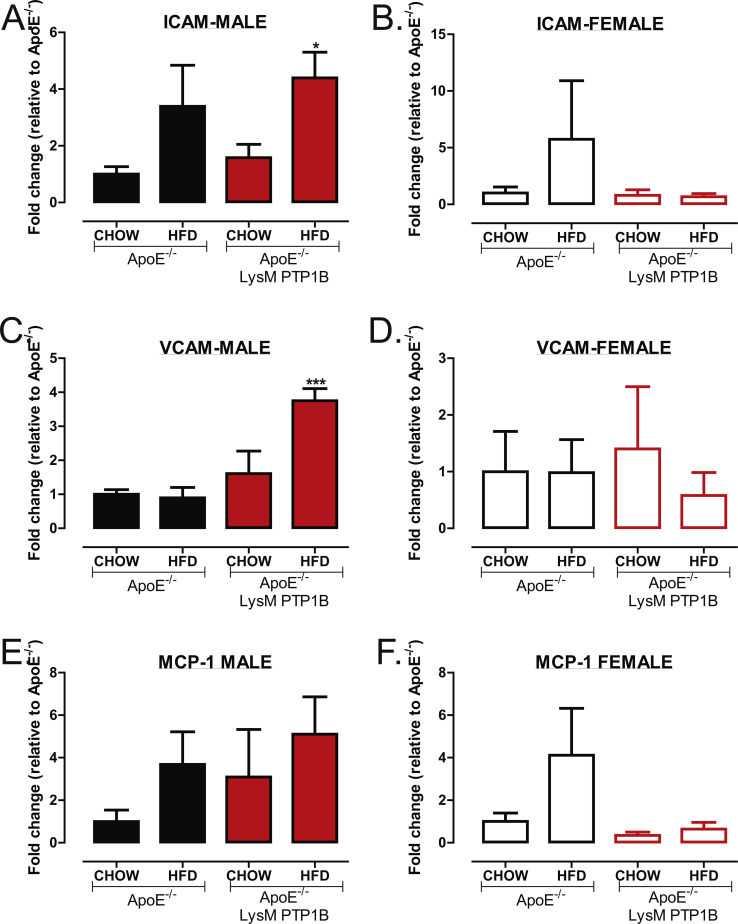


**Figure:**