# Supplementary material for: Myeloid protein tyrosine phosphatase 1B (PTP1B) deficiency protects against atherosclerotic plaque formation in the ApoE−/− mouse model of atherosclerosis with alterations in IL10/AMPKα pathway
Source: Mol Metab. 2017 Jun 13;6(8):845–53. doi: 10.1016/j.molmet.2017.06.003 (PMC5518727; doi:10.1016/j.molmet.2017.06.003)
Supplement: Supplementary file 1 [file mmc1.docx]

**Supplementary Materials and Methods**

**RNA extraction and qPCR.** Frozen tissues were lysed in Trizol reagent and RNA isolated using phenol/chloroform extraction. RNA was then synthesized into cDNA using tetrokits (Bioline) and subjected to qPCR analysis using SYBER and LightCycler 480 (Roche).

**Supplementary Figures Legends.**

**Supplementary Figure 1: Myeloid specific PTP1B deletion improves glucose maintenance independent of weight gain in aged CHOW mice.** Weights of male (**A**) and female (**B**) 6 month old ApoE^-/-^ and ApoE^-/-^/LysM-PTP1B CHOW fed mice. Glucose tolerance tests of male (**C**) and female (**D**) 6 month old CHOW cohorts. Mice were fasted for 5h prior to basal glucose monitoring (as described in materials and methods) and subsequently mice injected I.P. with 20% glucose (w/v) and blood re-analysed at 15, 30, 60 and 90 mins post-injection. Blood was collected at terminal culls and serum analysed for circulating insulin levels (**E, F**,using ELISA, Millipore). Data are represented as mean $\pm$ S.E.M. (n=7 per group) and analysed by unpaired two-tailed t-tests or two way ANOVA followed by Bonferonni multiple comparison t-tests where *p$\leq$0.05, **p$\leq$0.01, *** p$\leq$0.001, ****p$\leq$0.0001 when compared to ApoE^-/-^ control groups.

**Supplementary Figure 2: Myeloid specific PTP1B deletion reduces total cholesterol and triglycerides and increases circulating IL-10 in aged CHOW mice.**

Blood was collected at terminal culls and serum analysed for circulating total cholesterol (**A**, Sigma), triglycerides (**B**), IL-10 (**C**, ELISA, Millipore) or PGE_2_ (**D**, via lipidomics, UHI). For IL-10 analysis, values below the level of detection were designated 0. Data are represented as mean $\pm$ S.E.M. (n=4-7 per group) and analysed by unpaired two-tailed t-tests where *p$\leq$0.05, **p$\leq$0.01 when compared to ApoE^-/-^ control groups.

**Supplementary Figure 3: Genetic analysis of macrophage specific PTP1B deletion.** (**A-F**) Genetic analysis of aortic tissues (n=7 per group) as analysed by qPCR using SYBER and LightCycler 480 (Roche). Data are represented as mean $\pm$ S.E.M. and analysed by one way ANOVA followed by unpaired two-tailed t-tests where ***p$\leq$0.001 when compared to saline or ApoE^-/-^ CHOW control groups.

**Supplementary Figure 4: Western blot analysis of downstream IR and AMPK signalling pathways in myeloid specific PTP1B deletion mice.**

Western blot analysis of aortic tissues from male (**A,B**) and female (**F,G**) ApoE^-/-^ or ApoE^-/-^/LysM-PTP1B mice fed either CHOW (**A,F**) or HFD (**B,G**) and injected with either saline or insulin immediately prior to culling. Quantification of (**C,H**) p-S6 (Ser 235/236), (**D,I**) p-p38 (Thr 180/Tyr 182) and (**E,J**) total PTP1B of male and females represented in **A,B** and **F,G** respectively. Data are represented as mean $\pm$ S.E.M. and analysed by one way ANOVA followed by Dunnett’s post hoc analysis or unpaired two-tailed t-tests where *p$\leq$0.05, **p$\leq$0.01, ***p$\leq$0.01 when compared to saline or ApoE^-/-^ control groups
